# Germline mutations of the *STK11* gene in Korean Peutz–Jeghers syndrome patients

**DOI:** 10.1054/bjoc.1999.1125

**Published:** 2000-03-21

**Authors:** K-A Yoon, J-L Ku, H S Choi, S C Heo, S-Y Jeong, Y J Park, N K Kim, J C Kim, P M Jung, J-G Park

**Affiliations:** Korean Hereditary Tumor Registry, Laboratory of Cell Biology, Cancer Research Center and Cancer Research Institute; Department of Surgery, Seoul National University College of Medicine, 28 Yongon-dong, Seoul, Chongno-gu, 110-744, Korea; Department of Surgery, Yonsei University College of Medicine, Seoul, Korea; Department of Surgery, University of Ulsan College of Medicine; Pediatric Surgery Service, Hanyang University Hospital, Seoul, Korea

**Keywords:** Peutz–Jeghers syndrome, STK11, germline mutation

## Abstract

Peutz–Jeghers syndrome (PJS) is an autosomal dominantly inherited disease characterized by hamartomatous gastrointestinal polyps and mucocutaneous pigmentation, with an increased risk for various neoplasms, including gastrointestinal cancer. Recently, the PJS gene encoding the serine/threonine kinase STK11 (also named LKB1) was mapped to chromosome 19p13.3, and germline mutations were identified in PJS patients. We screened a total of ten Korean PJS patients (nine sporadic cases and one familial case including two patients) to investigate the germline mutations of the *STK11* gene. By polymerase chain reaction–single-strand conformation polymorphism and DNA sequencing analysis, three kinds of mis-sense mutation and a frame-shift mutation were identified: codon 232 (TCC to CCC) in exon 5, codon 256 (GAA to GCA) in exon 6, codon 324 (CCG to CTG) in exon 8, and a guanine insertion at codon 342 resulting in a premature stop codon in exon 8. These mis-sense variants were not detected in 100 control DNA samples. Furthermore, we found an intronic mutation at the dinucleotide sequence of a splice-acceptor site: a one base substitution from AG to CG in intron 1, which may cause aberrant splicing. Most reported germline mutations of the *STK11* gene in PJS patients were frame-shift or non-sense mutations resulting in truncated proteins. Together, these findings indicate that germline mis-sense mutations of the *STK11* gene are found in PJS patients in addition to truncating mutations. The effects of these mutations on protein function require further examination. In summary, we found germline mutations of the *STK11* gene in five out of ten Korean PJS patients. © 2000 Cancer Research Campaign


British Journal of Cancer (2000) **82**(8), 1403–1406
© 2000 Cancer Research Campaign
DOI: 10.1054/ bjoc.1999.1125, available online at http://www.idealibrary.com on IDEAL®

### Germline mutations of the *STK11* gene in Korean Peutz–Jeghers syndrome patients

**K-A Yoon[1], J-L Ku[1], HS Choi[2], SC Heo[2], S-Y Jeong[2], YJ Park[2], NK Kim[3], JC Kim[4], PM Jung[5] and J-G Park[1]**

[1]Korean Hereditary Tumor Registry, Laboratory of Cell Biology, Cancer Research Center and Cancer Research Institute, [2]Department of Surgery, Seoul National University College of Medicine, 28 Yongon-dong, Chongno-gu Seoul, 110-744, Korea; [3]Department of Surgery, Yonsei University College of Medicine, Seoul, Korea; [4]Department of Surgery, University of Ulsan College of Medicine, [5]Pediatric Surgery Service, Hanyang University Hospital, Seoul, Korea

**Summary** Peutz–Jeghers syndrome (PJS) is an autosomal dominantly inherited disease characterized by hamartomatous gastrointestinal polyps and mucocutaneous pigmentation, with an increased risk for various neoplasms, including gastrointestinal cancer. Recently, the PJS gene encoding the serine/threonine kinase STK11 (also named LKB1) was mapped to chromosome 19p13.3, and germline mutations were identified in PJS patients. We screened a total of ten Korean PJS patients (nine sporadic cases and one familial case including two patients) to investigate the germline mutations of the *STK11* gene. By polymerase chain reaction–single-strand conformation polymorphism and DNA sequencing analysis, three kinds of mis-sense mutation and a frame-shift mutation were identified: codon 232 (TCC to CCC) in exon 5, codon 256 (GAA to GCA) in exon 6, codon 324 (CCG to CTG) in exon 8, and a guanine insertion at codon 342 resulting in a premature stop codon in exon 8. These mis-sense variants were not detected in 100 control DNA samples. Furthermore, we found an intronic mutation at the dinucleotide sequence of a splice-acceptor site: a one base substitution from AG to CG in intron 1, which may cause aberrant splicing. Most reported germline mutations of the *STK11* gene in PJS patients were frame-shift or non-sense mutations resulting in truncated proteins. Together, these findings indicate that germline mis-sense mutations of the *STK11* gene are found in PJS patients in addition to truncating mutations. The effects of these mutations on protein function require further examination. In summary, we found germline mutations of the *STK11* gene in five out of ten Korean PJS patients. © 2000 Cancer Research Campaign

**Keywords:** Peutz–Jeghers syndrome; STK11; germline mutation

Peutz–Jeghers syndrome (PJS) is a disease of autosomal dominant inheritance that is characterized by hamartomatous gastrointestinal polyps and melanocytic pigmentation on the lips and perioral and buccal regions. Several studies have been reported on PJS associated with cancers of the gastrointestinal tract, pancreas, breast, ovary, uterine cervix and gallbladder (Giardiello et al, 1987; Spigelman et al, 1989). The PJS gene has been mapped to chromosome 19p13.3 by comparative genomic hybridization and linkage analysis in PJS patients (Hemminki et al, 1997). Recently, the *STK11* gene was identified, and truncating germline mutations of *STK11* have been reported in PJS patients (Hemminki et al, 1998;Jenne et al, 1998). The *STK11* gene is identical to the previously cloned but poorly characterized gene, *LKB1*. *LKB1* encodes a serine/threonine kinase with high homology to the Xenopus serine/threonine kinase XEEK1 (Su et al, 1996).

*STK11* extends over 23 kb, is composed of nine exons and is ubiquitously expressed in various adult human tissues. The fact that *STK11* is the first kinase-encoding gene associated with hereditary cancers which display inactivating germline mutations implies that the mutant proteins may affect the development of PJS phenotypes. Although the mutation rate of the *STK11* gene was low, somatic mutations in the *STK11* gene were detected in sporadic colorectal carcinomas, gastric carcinomas and malignant melanomas (Dong et al, 1998; Gruber et al, 1998; Park et al, 1998; Guldberg et al, 1999). Together, these reports suggest that *STK11* is a tumour suppressor gene and that genetic changes of *STK11* play an important role in the development of cancer as well as Peutz–Jeghers syndrome.

The present study screened a total of ten Korean PJS patients including nine sporadic cases and one familial case to investigate genetic alterations of the *STK11* gene.

*Received 13 August 1999*
*Revised 1 November 1999*
*Accepted 22 November 1999*

*Correspondence to:* J-G Park

### MATERIALS AND METHODS

### Patients

Mutational analysis of the *STK11* gene was performed with DNA samples from ten Korean PJS patients: SNU-P2, SNU-P5, SNU-P6, SNU-P7, SNU-P8, HYU-P2, HYU-P3, YSU-P5, USU-P1, USU-P2. Among these patients, SNU-P5 is a familial case including two PJS patients. For the family screening of SNU-P5, a brother and a sister of the patients were available. Other family members except the patients had no clinical signs of PJS. Clinical manifestations of the patients with PJS were shown in Table 1. In pathologic reports of all patients, hamartomatous polyps were proven. The number of polyps was ranged from 5 to 200. Mucocutaneous pigmentation was found in all patients. For genetic polymorphism studies, we screened for the *STK11* gene in DNA samples obtained from peripheral blood lymphocytes of 60 healthy people and in normal colonic mucosa DNA samples from 40 colorectal cancer patients.

**Table 1** Clinical manifestations of patients with Peutz–Jeghers syndrome

| Patient | Sex/Age | 1st symptom (age) | Mucocutaneous pigmentation | Distribution of polyp | Number of polyp | Family history | Laparotomy (× Number[a]) |
|---|---|---|---|---|---|---|---|
| SNU-P2 | M/49 | 8 | Yes | SB, CR | 45 | No | Yes (×3) |
| SNU-P5 | F/36 | 19 | Yes | SB, CR | 67 | Yes | Yes (×4) |
| | F/31 | 16 | Yes | SB, CR | ≅100 | Yes | Yes (×2) |
| SNU-P6 | M/23 | 18 | Yes | ST, SB, CR | 50 | No | Yes (×1) |
| SNU-P7 | M/31 | 12 | Yes | SB, CR | 30 | No | Yes (×3) |
| SNU-P8 | F/28 | 25 | Yes | ST, SB, CR | 10 | No | Yes (×1) |
| HYU-P2 | F/25 | 21 | Yes | ST, SB, CR | 100–200 | No | Yes (×2) |
| HYU-P3 | M/26 | 11 | Yes | ST, SB, CR | 100–200 | No | Yes (×2) |
| YSU-P5 | F/28 | 22 | Yes | ST, SB | ≅100 | No | Yes (×2) |
| USU-P1 | M/22 | 16 | Yes | SB, CR | 5 | No | No |
| USU-P2 | F/29 | 15 | Yes | ST, SB, CR | ≅100 | No | Yes (×2) |

M, male; F, female; ST, stomach; SB, small bowel; CR, colorectum. [a]Endoscopic polypectomy was not included.

### DNA extraction

Peripheral blood lymphocytes were isolated using Ficoll-Paque according to the manufacturer's instructions (Pharmacia Biotech, Uppsala, Sweden). Total genomic DNA of lymphocytes was extracted using TRI reagent according to the manufacturer's instructions (Molecular Research Center, Cincinnati, OH, USA). Normal colonic mucosa DNAs were extracted according to the standard sodium dodecyl sulphate (SDS)–proteinase K procedure.

### PCR amplification and single strand conformation polymorphism

The polymerase chain reaction (PCR) primer pairs were used as described by Jenne et al (1998). PCR reactions contained, in a final reaction volume of 25 μl, 100 ng of genomic DNA, 2.5 pmoles of each primer, 250 μM of each dNTPs, and 0.5 units of *Taq* DNA polymerase. The PCR reaction buffer and Q solution were provided by the supplier (Qiagen, Hilden, Germany). PCR reactions were initiated by denaturing the DNA for 5 min at 94°C in a programmable thermal cycler (Perkin-Elmer Cetus 9600: Roche Molecular Systems, Inc., NJ, USA). PCR cycles were: 35 cycles at 94°C for 30 s, 62°C for 1 min, and 72°C for 1 min, with a final elongation for 10 min at 72°C.

For single-strand conformation polymorphism (SSCP), the genomic DNA was amplified in a final volume of 10 μl. Each exon of the *STK11* gene underwent the same PCR procedure as described above, with the addition of [α-$^{32}$P]-dCTP (Amersham, Arlington Heights, IL, USA). The region from exon 4 to exon 5 was amplified in a single PCR reaction and then digested with *Mae*III endonuclease (Boehringer Mannheim, Germany) at 55°C for 2 h, whereas exons 1, 2, 3, 6, 7, 8 and 9 were amplified separately.

Radiolabelled PCR reaction products were mixed with 95% formamide dye, denatured at 94°C for 5 min and chilled on ice. Three microlitres of each mixture was loaded onto a non-denaturing SSCP gel (6% polyacrylamide gel (19:1) with 10% glycerol in 1 × TBE buffer) and separated for 12–16 h at room temperature at a constant 300 V. After electrophoresis, the gel was transferred to 3MM Whatman paper, dried on a gel dryer and subjected to autoradiography.

### Cloning and sequencing

Samples showing abnormal bands by SSCP were subjected to cloning for DNA sequencing analysis. Fresh PCR products were ligated into pCR®-TOPO vectors and subcloned using the TA cloning system (Invitrogen, San Diego, CA, USA). A minimum of ten individual colonies were selected and cultured overnight in LB medium containing 50 μg ml$^{-1}$ ampicillin. Plasmid DNAs were isolated and used for DNA sequencing analysis. Bi-directional sequencing analysis was performed using either the dideoxy chain termination method with a T7 DNA polymerase sequencing kit (Pharmacia Biotech Inc., Piscataway, NJ, USA) or the Taq dideoxy terminator cycle sequencing kit on an ABI 377 DNA sequencer (Perkin-Elmer, Foster City, CA, USA). Sequences of target DNA were determined by using the original PCR primers.

### RESULTS

To investigate the genetic alteration of the *STK11* gene, we screened nine exons by PCR-SSCP analysis in ten Korean PJS patients. PCR-SSCP analysis revealed abnormal band shifts in exon 5 in two patients (SNU-P5 and her sister). However, the analysis revealed no abnormal bands in other samples, including two normal family members of the SNU-P5. In exon 6, the SSCP pattern of the SNU-P2 showed a different band pattern compared to that of other patients and controls. In exon 8, abnormal SSCP patterns were found in two unrelated patients (the SNU-P6 and the HYU-P2). An abnormal SSCP band pattern in exon 2 was detected in the USU-P2.

Sequencing analysis revealed three mis-sense mutations and a frame-shift mutation in exons 5, 6 and 8. The SNU-P5 and her sister had the same mis-sense mutation from TCC (Ser) to CCC (Pro) at codon 232 in exon 5 (Figure 1A). This mutation was not detected in other family members of SNU-P5. Two missense mutations from GAA (Glu) to GCA (Ala) at codon 256 in exon 6 and from CCG (Pro) to CTG (Leu) at codon 324 in exon 8 were identified in the SNU-P2 and the HYU-P2 respectively (Figure 1B). These mis-sense variants were not detected in DNA samples from peripheral blood lymphocytes of 60 healthy people or in normal colonic mucosa DNA from 40 colorectal cancer patients. The SNU-P6 exhibited a frame-shift mutation by insertion of an additional guanine at codon 342, resulting in a premature stop codon in exon 8 (Figure 2). In the USU-P2, we found an intronic mutation at a splice-acceptor sequence. The transversion from the dinucleotide sequence AG to CG in intron 1 would theoretically cause aberrant splicing that may result in a truncated protein. However, we could not confirm the occurrence of aberrant

*© 2000 Cancer Research Campaign*

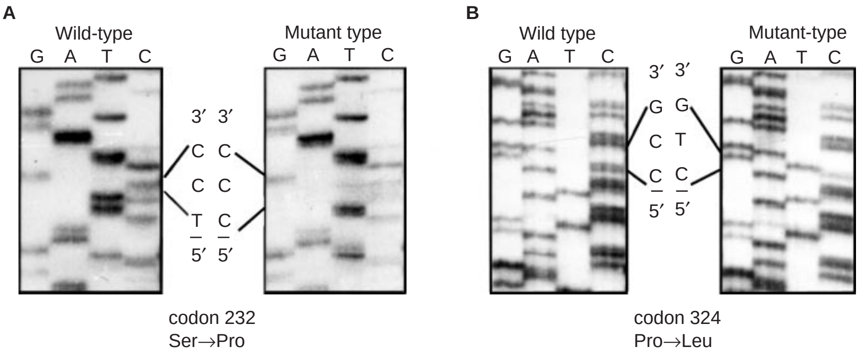

**Figure 1** The mis-sense mutations of the *STK11* gene in the germline. Mis-sense mutations in exon 5 of two patients (the SNU-P5 and her sister) (**A**) and exon 8 of the HYU-P2 (**B**)

**Table 2** Germline mutations of the *STK11* gene in Korean PJS families

| Patient | Location | Codon | Nucleotide change | Predicted effect |
|---|---|---|---|---|
| SNU-P2 | Exon 6 | 256 | GAA→GCA | Glu→Ala |
| SNU-P5 | Exon 5 | 232 | TCC→CCC | Ser→Pro[a] |
| SNU-P6 | Exon 8 | 342 | G insertion GAG→GGAG | Premature stop at codon 359 |
| HYU-P2 | Exon 8 | 324 | CCG→CTG | Pro→Leu |
| USU-P2 | Intron 1 | | ccagGG→cccgGG | Aberrant splicing? |

[a]Two patients (SNU-P5 and her sister) shared the same mutation in exon 5 of the *STK11* gene.

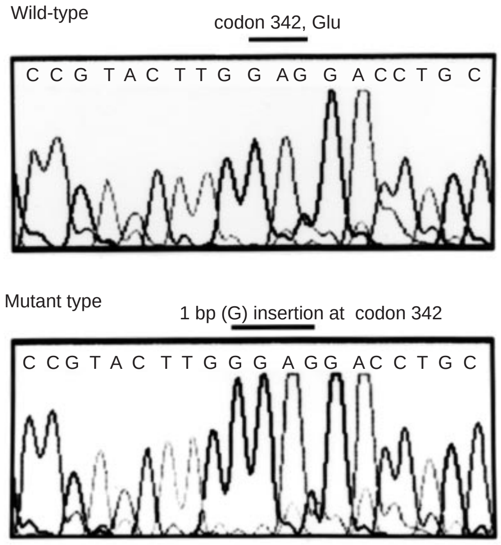

**Figure 2** The frame-shift mutation of the *STK11* gene in the germline. Frame-shift mutation in exon 8 of *STK11* of the SNU-P6. The guanine insertion at codon 342 results in a truncating mutation at codon 359

splicing due to a lack of available mRNA. The results of these mutational analyses are summarized in Table 2.

### DISCUSSION

Three mis-sense mutations, a frame-shift mutation and an intronic mutation at a splice-acceptor site were identified in five out of the ten Korean PJS patients we examined. Most germline mutations reported of the *STK11* gene in PJS patients were either frame-shift or non-sense mutations resulting in truncated proteins (Gruber et al, 1998; Hemminki et al, 1998; Jenne et al, 1998; Nakagawa et al, 1998). All mutations of *STK11* that lead to truncated proteins with incomplete catalytic domains are unlikely to exhibit kinase activity (Jenne et al, 1998). The SNU-P6 exhibited a frame-shift mutation of *STK11* which resulted in a premature stop codon in exon 8. The USU-P2 showed an intronic mutation at a splice-acceptor site in intron 1 that may cause aberrant splicing and a truncated protein. In this splice acceptor site, three kinds of germline mutation were reported in PJS patients by other groups (Nakagawa et al, 1998; Westerman et al, 1999). Therefore, STK11 is thought to lose its serine/threonine kinase function due to a truncated gene product, promoting the development of the PJS phenotype.

Three of the five mutations we detected were mis-sense mutations. Only 16% of previously reported germline mutations in *STK11* in PJS patients were of the mis-sense type, although mis-sense mutations have been detected in sporadic colon and stomach cancers (Dong et al, 1998; Park et al, 1998; Hemminki, 1999). The three mis-sense mutations of *STK11* we found occurred in codons 232, 256 and 324. Codons 37–314 of *STK11* share 93% homology with Xenopus early embryonic kinase 1, XEEK1 (GenBank accession no. U24435) and 96% homology with mouse Lkb1 (GenBank accession no. AF145287), as well as these three codons (232, 256, 324) are identical with those of XEEK1 and mouse Lkb1 (Smith et al, 1999). Moreover, the catalytic core of the presumed kinase domain of STK11 is located between codons 50 and 337 (Hemminki et al, 1998; Jenne et al, 1998). Therefore, we suspect that these mis-sense mutations we found could affect the function of STK11 through alteration of the kinase domain. Among the family members of the SNU-P5, her sister is another PJS patient. Because these two sisters shared the same mutation in exon 5 of

© 2000 Cancer Research Campaign 

the *STK11* gene, we concluded that this mis-sense mutation exhibited penetrance in these patients. To exclude the possibility that these mutations were genetic polymorphisms, we screened exons 5, 6 and 8 of *STK11* in blood DNA samples of 60 healthy people and in normal mucosal tissue DNA from 40 colon cancer patients. Because the results of SSCP in these controls proved to be normal, the mutations detected in PJS patients were not considered polymorphic variants but germline mutations or rare polymorphisms of the *STK11* gene. The effects of these mis-sense mutations on *STK11* functional activity require further examination, and are the subject of future studies.

Members of PJS families are at risk of developing Peutz–Jeghers syndrome. Thus, it may be more feasible to offer predictive genetic testing to members of PJS families instead of repeated physical examinations (Nakagawa et al, 1998). Five out of ten Korean PJS patients we examined had germline mutations of the *STK11* gene. Owing to the high rate of germline mutation in the *STK11* gene in PJS patients, screening for *STK11* mutations in PJS patients should be used as a diagnostic tool.

### ACKNOWLEDGEMENTS

We thank Sir Walter Bodmer and Dr Ian Tomlinson for helpful discussion and critical review of this manuscript. This work was supported in part by a grant from the Korea Science and Engineering Foundation (KOSEF) through the Cancer Research Center at Seoul National University (KOSEF-CRC-97-8).

### REFERENCES

Dong SM, Kim KM, Kim SY, Shin MS, Na EY, Lee SH, Park WS, Yoo NJ, Jang JJ, Yoon CY, Kim JW, Kim SY, Yang YM, Kim SH, Kim CS and Lee JY (1998) Frequent somatic mutations in serine/threonine kinase 11/Peutz–Jeghers syndrome gene in left-sided colon cancer. *Cancer Res* **58**: 3787–3790

Giardiello FM, Welsh SB, Hamilton SR, Offerhaus GJ, Gittelsohn AM, Booker SV, Krush AJ, Yardley JH and Luk GD (1987) Increased risk of cancer in the Peutz–Jeghers syndrome. *N Engl J Med* **11**: 1511–1514

Gruber SB, Entius MM, Petersen GM, Laken SJ, Longo PA, Boyer R, Levin AM, Mujumda UJ, Trent JM, Kinzler KW, Vogelstein B, Hamilton SR, Polymeropoulos MH, Offerhaus GJ and Giardiello FM (1998) Pathogenesis of adenocarcinoma in Peutz–Jeghers syndrome. *Cancer Res* **58**: 5267–5270

Guldberg P, thor Straten P, Ahrenkiel V, Seremet T, Kirkin AF and Zeuthen J (1999) Somatic mutation of the Peutz–Jeghers syndrome gene, LKB1/STK11, in malignant melanoma. *Oncogene* **18**: 1777–1780

Hemminki A (1999) The molecular basis and clinical aspects of Peutz–Jeghers syndrome. *Cell Mol Life Sci* **55**: 735–750

Hemminki A, Tomlinson I, Markie D, Jarvinen H, Sistonen P, Bjorkqvist AM, Knuutila S, Salovaara R, Bodmer W, Shibata D, Chapelle A and Aaltonen LA (1997) Localization of a susceptibility locus for Peutz–Jeghers syndrome to 19p using comparative genomic hybridization and targeted linkage analysis. *Nat Genet* **15**: 87–90

Hemminki A, Markie D, Tomlinson I, Avizienyte E, Roth S, Loukola A, Bignell G, Warren W, Aminoff M, Hoglund P, Jarvinen H, Kristo P, Pelin K, Ridanpaa M, Salovaara R, Toro T, Bodmer W, Olschwang S, Olsen AS, Stratton MR, Chapelle A and Aaltonen LA (1998) A serine/threonine kinase gene defective in Peutz–Jeghers syndrome. *Nature* **391**: 184–187

Jenne DE, Reimann H, Nezu J, Friedel W, Loff S, Jeschke R, Muller O, Back W and Zimmer M (1998) Peutz–Jeghers syndrome is caused by mutations in a novel serine threonine kinase. *Nat Genet* **18**: 38–44

Nakagawa H, Koyama K, Miyoshi Y, Ando H, Baba S, Watatani M, Monden M and Nakamura Y (1998) Nine novel germline mutations of STK11 in ten families with Peutz–Jeghers syndrome. *Hum Genet* **103**: 168–172

Park WS, Moon YW, Yang YM, Kim YS, Kim YD, Fuller BG, Vortmeyer AO, Fogt F, Lubensky IA and Zhuang Z (1998) Mutations of the STK11 gene in sporadic gastric carcinoma. *Int J Oncol* **13**: 601–604

Smith DP, Spicer J, Smith A, Swift S and Ashworth A (1999) The mouse Peutz–Jeghers syndrome gene Lkb1 encodes a nuclear protein kinase. *Hum Mol Genet* **8**: 1479–1485

Spigelman AD, Murday V and Phillips RK (1989) Cancer and the Peutz–Jeghers syndrome. *Gut* **30**: 1588–1590

Su JY, Erikson E and Maller JL (1996) Cloning and characterization of a novel serine/threonine protein kinase expressed in early Xenopus embryos. *J Biol Chem* **271**: 14430–14437

Westerman AM, Entius MM, Boor PPC, Koole R, Baar E, Offerhaus GJA, Lubinski J, Lindhout D, Halley DJJ, Rooij FWM and Wilson JHP (1999) Novel mutations in the LKB1/STK11 gene in Dutch Peutz–Jeghers families. *Hum Mutat* **13**: 476–481

 *© 2000 Cancer Research Campaign*
